# Data on the detail information of influence of substrate temperature on the film morphology and photovoltaic performance of non-fullerene organic solar cells

**DOI:** 10.1016/j.dib.2017.07.065

**Published:** 2017-07-27

**Authors:** Jicheng Zhang, SuFei Xie, Zhen Lu, Yang Wu, Hongmei Xiao, Xuejuan Zhang, Guangwu Li, Cuihong Li, Xuebo Chen, Wei Ma, Zhishan Bo

**Affiliations:** a Beijing Key Laboratory of Energy Conversion and Storage Materials, College of Chemistry, Beijing Normal University, No.19, Xinjiekouwai Street, Haidian District, Beijing 100875, China; b College of Chemistry and Environmental Engineering, ShanXi DaTong University, Datong 037009, China; c State Key Laboratory for Mechanical Behavior of Materials, Xi'an Jiaotong University, Xi'an 710049, China

## Abstract

This data contains additional data related to the article “Influence of Substrate Temperature on the Film Morphology and Photovoltaic Performance of Non-fullerene Organic Solar Cells” (Jicheng Zhang et al., In press) [1]. Data include measurement and characterization instruments and condition, detail condition to fabricate norfullerene solar cell devices, hole-only and electron-only devices. Detail condition about how to control the film morphology of devices via tuning the temperature of substrates was also displayed. More information and more convincing data about the change of film morphology for active layers fabricated from different temperature, which is attached to the research article of “Influence of Substrate Temperature on the Film Morphology and Photovoltaic Performance of Non-fullerene Organic Solar Cells” was given.

**Specifications Table**

**Table:** 

Subject area	Chemistry
More specific subject area	Non-fullerene acceptors for organic solar cells
Type of data	image, text file, figure
How data was acquired	^1^H and ^13^C NMR spectra were recorded on a Bruker AV 400 spectrometer. UV-visible absorption spectra were measured on a PerkinElmer UV–vis spectrometer model Lambda 750. Atomic force microscopy (AFM) measurements were conducted under ambient conditions using a Digital Instrument Multimode Nanoscope IIIA using the tapping mode. The thickness of the blend films was measured by a Dektak 6 M surface profilometer. XRD experiments were performed with an X' Pert PRO MPD instrument. The electrochemical behaviour of the polymers was studied using cyclic voltammetry (CHI 630 A Electrochemical Analyzer) with a three-electrode electrochemical cell in a 0.1 M Bu4NPF6 CH3CN solution under an atmosphere of nitrogen with a scanning rate of 0.1 V/s. A glassy carbon working electrode, a Pt wire counter electrode and an Ag/AgNO_3_ (0.01 M in CH3CN) reference electrode were used. The ferrocene/ferrocenium (Fc/Fc+) redox couple was used as the internal reference standard. Current-voltage (I-V) and external quantum efficiency (EQE) measurements were conducted in air without encapsulation. The I-V characteristics were recorded at room temperature using an Agilent B2902A Source Meter under the illumination of an AM1.5 G AAA class solar simulator (model XES-301S, SAN-EI) with an intensity of 100 mW cm^*−2*^, and the white light intensity was calibrated with a standard single-crystal Si solar cell.
Data format	analyzed
Experimental factors	A mixture of **PCDTBT-C12** and **NI-T-C8** in 1,2-dichlorobenzene (DCB) was firstly stirred at 90 °C overnight to ensure sufficient dissolution. To achieve a purpose substrate temperature, the substrates and micro pipette tips were elevated to 30 or 45 ^o^C by storage on a hotplate for 5 min, then the donor/acceptor mixed solutions were quickly pipetted to a heated substrate and spin-coated to form an active layer.
Experimental features	Tuning the aggregation of small molecular acceptors when blended with donor materials via control the temperature of substrates.
Data source location	Beijing Normal University of China, Beijing, China
Data accessibility	Data is within this article.

**Value of the data**

• Detail information about how to control the temperature of substrates to tune the aggregation of acceptors in the active layers.
• Give more convincing data for the film morphology analysis of blend films fabricated from substrates with different temperature.
• Give detail information about how to fabricate non fullerene solar cells, hole-only and electron-only devices.

## 1 Data

**Fig. 1:**
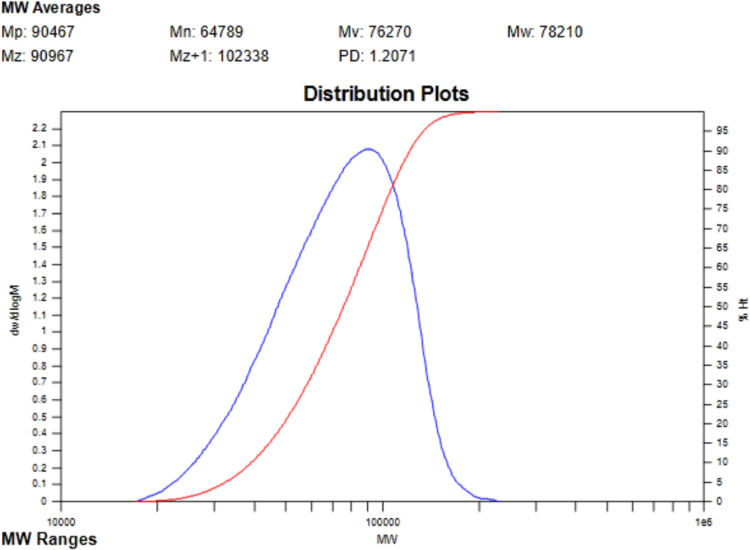
GPC images of **PCDTBT-C12**.

**Fig. 2:**
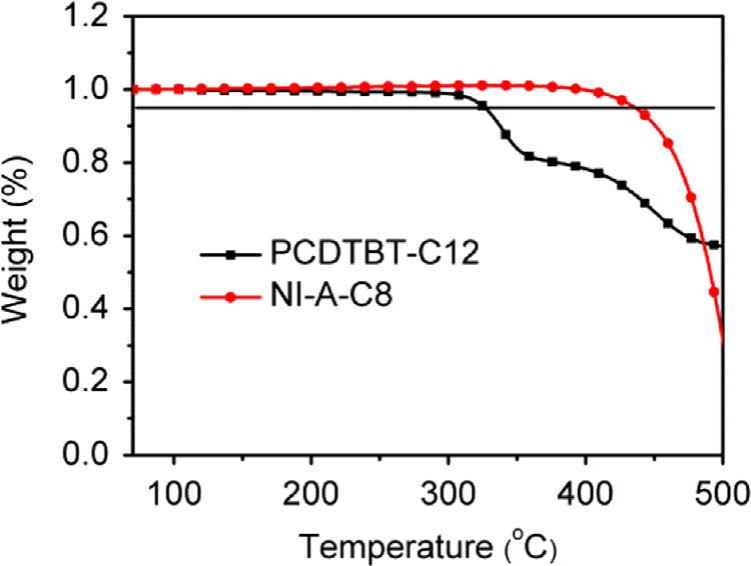
TGA images of **PCDTBT-C12** and **NI-A-C8**.

**Fig. 3:**
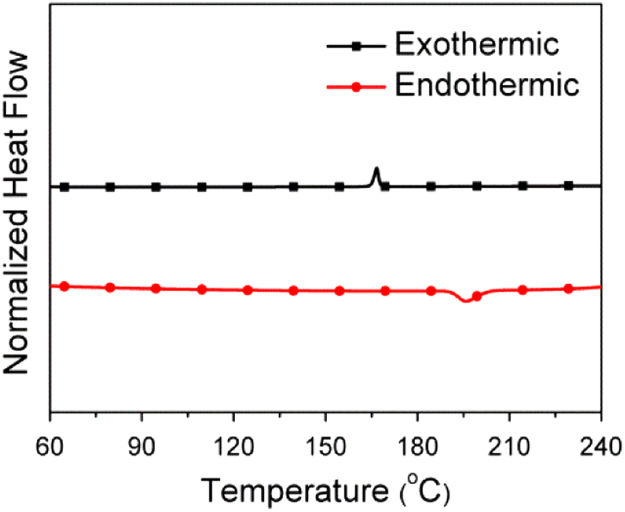
DSC images of **NI-A-C8**.

**Fig. 4:**
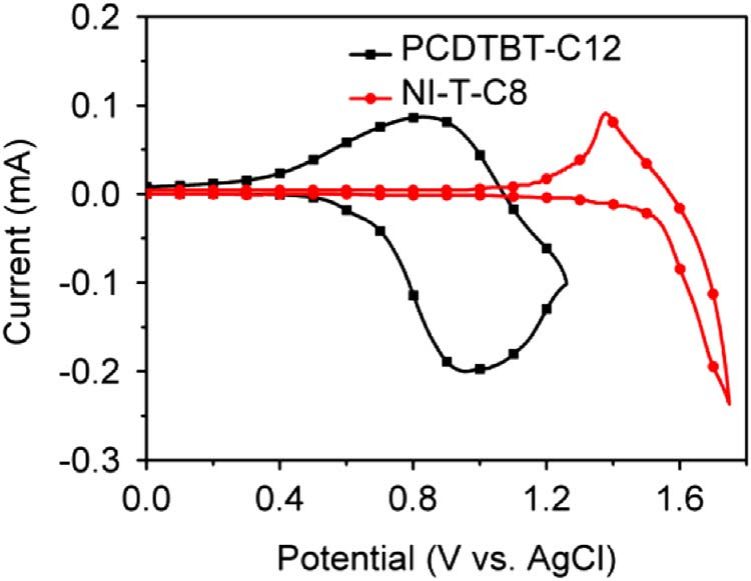
Cyclic voltammetry curves of **PCDTBT-C12 and NI-T-C8**.

**Fig. 5:**
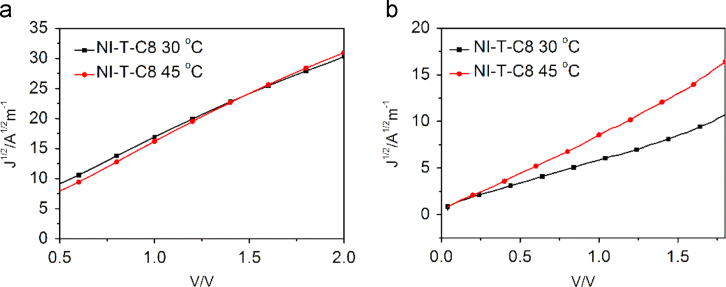
*J*^1/2^–*V* curves of **PCDTBT-C12:NI-T-C8** based devices for determining *μ*_*h*_ and *μ*_*e*_. (a) *μ*_*h*_ and (b) *μ*_*e*_ of active layers spin-coated on substrates with different temperature.

**Fig. 6:**
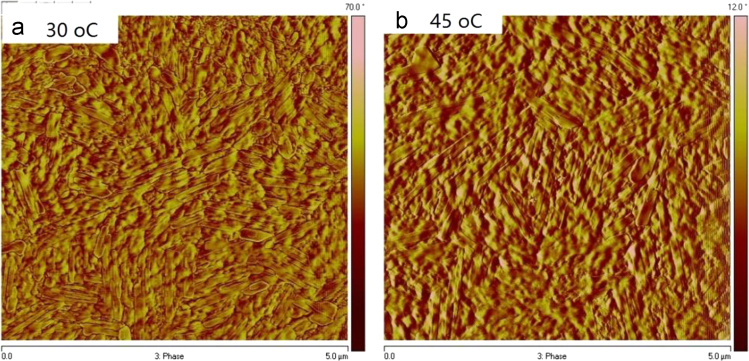
Phase images of **PCDTBT-C12:NI-T-C8** blend films fabricated from substrates of 30 °C (a) and 45 °C (b).

**Fig. 7:**
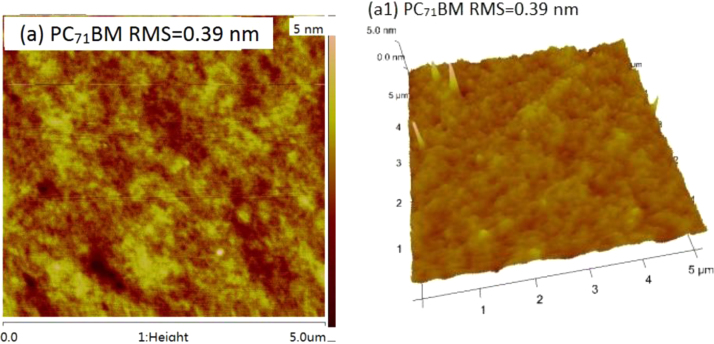
AFM height images (a) and three-dimensional images (a1, 5 μm × 5 μm) of **PCDTBT-C12:PC**_**71**_**BM** blend films fabricated under optimized conditions.

**Fig. 8:**
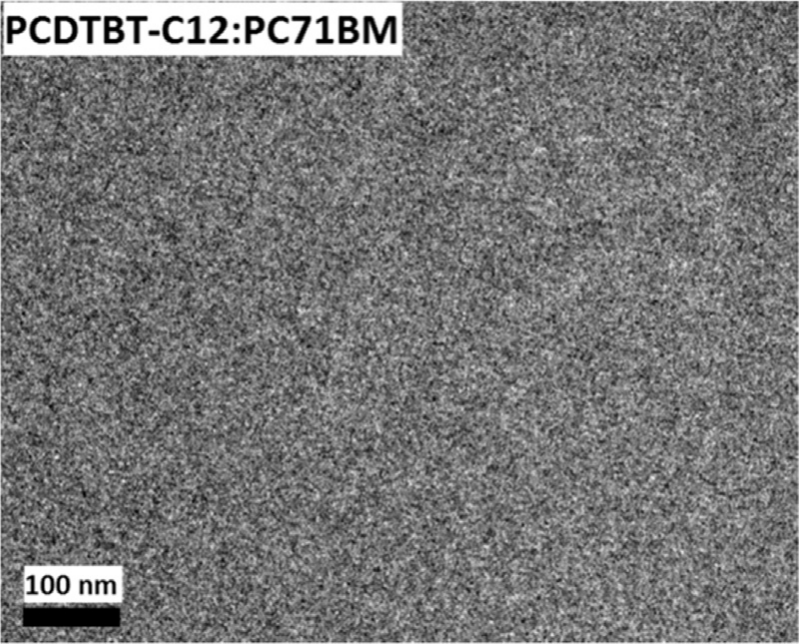
TEM images **PCDTBT-C12:PC**_**71**_**BM** blend films fabricated under optimized conditions.

The data include GPC results of ***PCDTBT-C12***,(Fig. 1) TGA images of ***PCDTBT-C12*** (Fig. 2)and ***NI-A-C8*,** DSC images of ***NI-A-C8***,(Fig. 3) Cyclic voltammetry curves (Fig. 4)of ***PCDTBT-C12*** and ***NI-T-C8***, *J*^1/2^–*V* curves of ***PCDTBT-C12:NI-T-C8*** based devices for determining *μ_h_* and *μ_e_*,(Fig. 5) Phase images of ***PCDTBT-C12:NI-T-C8*** blend films fabricated from substrates of 30 °C and 45 °C(Fig. 6, Fig. 7, Fig. 8), AFM height images and three-dimensional images of ***PCDTBT-C12:PC**_**71**_**BM*** blend films fabricated under optimized conditions, TEM images ***PCDTBT-C12:PC**_**71**_**BM*** blend films fabricated under optimized conditions*.*

## 2 Experimental design, materials and methods

### 2.1 Measurements and characterization

Unless otherwise noted, all reactions were performed under a nitrogen atmosphere and were monitored by thin layer chromatography (TLC) on silica gel plates. ^1^H and ^13^C NMR spectra were recorded on a Bruker AV 400 spectrometer. UV-visible absorption spectra were measured on a PerkinElmer UV–vis spectrometer model Lambda 750. Atomic force microscopy (AFM) measurements were conducted under ambient conditions using a Digital Instrument Multimode Nanoscope IIIA using the tapping mode. The thickness of the blend films was measured by a Dektak 6 M surface profilometer. XRD experiments were performed with an X' Pert PRO MPD instrument. The electrochemical behaviour of the polymers was studied using cyclic voltammetry (CHI 630A Electrochemical Analyzer) with a three-electrode electrochemical cell in a 0.1 M Bu4NPF6 CH3CN solution under an atmosphere of nitrogen with a scanning rate of 0.1 V/s. A glassy carbon working electrode, a Pt wire counter electrode and an Ag/AgNO_3_ (0.01 M in CH3CN) reference electrode were used. The ferrocene/ferrocenium (Fc/Fc+) redox couple was used as the internal reference standard.

### 2.2 Organic solar cells fabrication and characterization

OSCs were fabricated with the device configuration of ITO/PEDOT:PSS (35 nm)/ **PCDTBT-C12:NI-T-C8**/LiF (0.7 nm)/Al (100 nm). The conductivity of ITO is 15 Ω. PEDOT:PSS (Baytron Al 4083 from H.C. Starck) was filtered with a 0.45 mm polyvinylidene difluoride (PVDF) film before use. A PEDOT:PSS thin layer was spin-coated on top of the cleaned ITO substrate at 3000 rpm/s for 50 s and was dried at 130 °C for 20 min on a hotplate. The thickness of the PEDOT:PSS layer was approximately 35 nm. A mixture of **PCDTBT-C12** and **NI-T-C8** in 1,2-dichlorobenzene (DCB) was firstly stirred at 90 °C overnight to ensure sufficient dissolution. To achieve a purpose substrate temperature, the substrates and micro pipette tips were elevated to 30 or 45 ^*o*^C by storage on a hotplate for 5 min, then the donor/acceptor mixed solutions were quickly pipetted to a heated substrate and spin-coated to form an active layer. The concentrations of the blend of **PCDTBT-C12** and small molecules were all 25 mg/mL. A top electrode of 0.7 nm LiF and 100 nm of aluminium were thermally evaporated at a pressure of 10^−4^ Pa through a shadow mask. On one substrate, five cells with an effective area of 0.04 cm^*2*^ each were fabricated. Current-voltage (I-V) and external quantum efficiency (EQE) measurements were conducted in air without encapsulation. The I-V characteristics were recorded at room temperature using an Agilent B2902A Source Meter under the illumination of an AM1.5 G AAA class solar simulator (model XES-301S, SAN-EI) with an intensity of 100 mW cm^−2^, and the white light intensity was calibrated with a standard single-crystal Si solar cell.

### 2.3 Space-charge limited current measurement

Hole-only devices with a structure of ITO/PEDOT:PSS (35 nm)/***PCDTBT-C12:NI-T-C8**/Au* (100 nm) and electron-only devices with a configuration of FTO/***PCDTBT-C12:NI-T-C8***/Al (100 nm) were fabricated. FTO substrates were prepared by etching the commercial FTO substrates with HCl and Zn powders. The blend solution of ***PCDTBT-C12*** and ***NI-T-C8*** in DCB was spin-coated onto the PEDOT:PSS layer to form the active layer, like OSC devices, and 100 nm of Au was thermally evaporated at a pressure of 10^−4^ Pa through a shadow mask. For electron-only devices, the blend solution of ***PCDTBT-C12*** and ***NI-T-C8*** in DCB was spin-coated on the clean FTO substrates to form an active layer. Al electrodes (100 nm) were vacuum-deposited on the polymer thin films. Dark *J–V* curves of the hole-only devices and electron-only devices were measured by the space-charge limited current (SCLC) method. The dark *J*–*V* curves of the devices were fitted using the Mott–Gurney equation: *J* = 9*ε*_*o*_*ε*_*r*_*μV*^*2*^*/8L*^*3*^, where *J* is the space-charge limited current, *ε*_*o*_ is the vacuum permittivity, *ε*_*r*_ is the permittivity of the active layer, μ is the hole mobility or the electron mobility, and L is the thickness of the active layer (Fig. 5).
